# Histotripsy for multifocal breast cancer liver metastases with early complete metabolic response: a case report

**DOI:** 10.3389/fonc.2026.1782397

**Published:** 2026-04-07

**Authors:** Tiffany M. Juarez, Victoria Chuchua, Kevin Burns

**Affiliations:** 1 Providence Mission Hospital, Leonard Cancer Institute, Mission Viejo, CA, United States; 2 Providence Mission Hospital, Department of Interventional Radiology, Mission Viejo, CA, United States

**Keywords:** breast cancer, Edison System, histotripsy, liver cancer, locoregional, metastases

## Abstract

Histotripsy is a non−thermal, non−ionizing focused ultrasound therapy that mechanically disrupts tissue through acoustic cavitation. We present a case of a woman with estrogen receptor–positive metastatic breast cancer who developed progressive multifocal liver metastases despite having failed multiple lines of systemic therapies. She underwent two histotripsy treatments staged approximately six weeks apart, using a lobar approach to sequentially treat left- and right-lobe lesions, targeting a total of 10 hepatic lesions. She tolerated both procedures well without any complications or interruption of her targeted therapy (trastuzumab deruxtecan). Follow-up PET/CT performed two months after the second treatment demonstrated complete metabolic response in the liver and overall disease regression, which was again observed on a subsequent PET/CT performed six months later. Follow-up MR imaging four and seven months after the second histotripsy revealed expected post−treatment involution of the treatment zones. This case highlights the ability of histotripsy to treat patients with advanced liver metastases in a staged fashion to minimize potential toxicities. These encouraging early results support the need for more data to confirm the role of histotripsy in advanced metastatic breast cancer patients who have limited treatment options.

## Introduction

Although the prognosis for patients with metastatic breast cancer has improved over the last decade with the advent of molecularly targeted drug therapy, management remains challenging. The growing number of systemic treatment options has extended median survival to almost 30 months. However, the resistance of cancer cells to such targeted agents limits their effectiveness thereby shortening periods of disease control (1). When the liver is the dominant site of metastatic disease, management becomes particularly challenging. In such cases, systemic therapy alone often fails to achieve durable control, and progressive hepatic disease can lead to liver failure, precluding further treatment and significantly impacting survival. Yet, current National Comprehensive Cancer Network (NCCN) guidelines offer limited recommendations for locoregional interventions in breast cancer, leaving a critical gap for patients with significant hepatic tumor burden (2). In this context, liver-directed therapies can play a pivotal role by slowing or halting liver progression while preserving liver function, minimizing treatment-related toxicities and improving quality of life (3).

Histotripsy is one such emerging non-invasive ablative therapy that employs short, high−amplitude ultrasound pulses to generate a cavitation “bubble cloud” that liquefies tissue at the focus without heat or ionizing radiation (4, 5). The #HOPE4LIVER clinical trial established treatment efficacy combined with a low toxicity profile which led to regulatory approval of histotripsy for the treatment of liver tumors by the FDA (6–8). Follow-up data at 1-year demonstrated 90% local tumor control (9). Early multicenter experiences outside the context of a clinical trial confirmed the high technical success and favorable safety in liver tumors (10, 11). Histotripsy provides precise tumor destruction with minimal risk to surrounding healthy tissues and, because of its unique mechanism of action, does not cause either thermal or radiation-induced toxicities. Moreover, unlike other locoregional therapies, it treats liver tumors irrespective of their blood supply and destroys them instantaneously. Another benefit of histotripsy is that systemic therapy does not need to be interrupted. Here we describe a patient with metastatic breast cancer who received two sessions of histotripsy in a staged fashion without interruption of her systemic targeted therapy which ultimately led to a complete metabolic response as demonstrated on PET imaging.

## Case report

A 30-year-old woman with estrogen receptor–positive (ER+) invasive ductal carcinoma initially underwent lumpectomy in 2011, followed by adjuvant multiagent chemotherapy, radiation, and endocrine therapy. She developed metastatic recurrence in September 2016 involving the lung, bone, and several lymph nodes. Her treatment strategy evolved over several years, reflecting the progression of her disease and receptor heterogeneity noted in serial biopsies. These biopsies showed varying levels of ER, PR, and HER2 expression, indicating the dynamic nature of her tumor biology. Starting in October 2016, she was treated with a combination of fulvestrant, palbociclib, and denosumab. However, by May 2021, imaging revealed increased osseous lesions, leading to palliative sacral radiotherapy and initiation of capecitabine. Liver metastases developed in January 2023 prompting treatment with everolimus and exemestane in March 2023. Nonetheless, by November 2023, she again progressed both in the bones and liver, triggering another change in therapy to capivasertib and fulvestrant in December 2023. In January 2024, she underwent palliative radiation to the lumbar spine for symptom relief. Continued disease progression led to further adjustments in her treatment plan, with elacestrant being added to her regimen in September 2024 and abemaciclib in December 2024. New hepatic lesions identified by CT imaging in March 2025were considered unlikely to be benign due to their rapid development, multiplicity, and imaging features consistent with disease progression. There were no financial, cultural, or access-to-testing barriers that limited the patient’s ability to obtain diagnostic imaging or laboratory evaluation throughout her care. These factors made metastatic involvement the most probable explanation thereby prompting treatment with trastuzumab deruxtecan.

**Figure 1:**
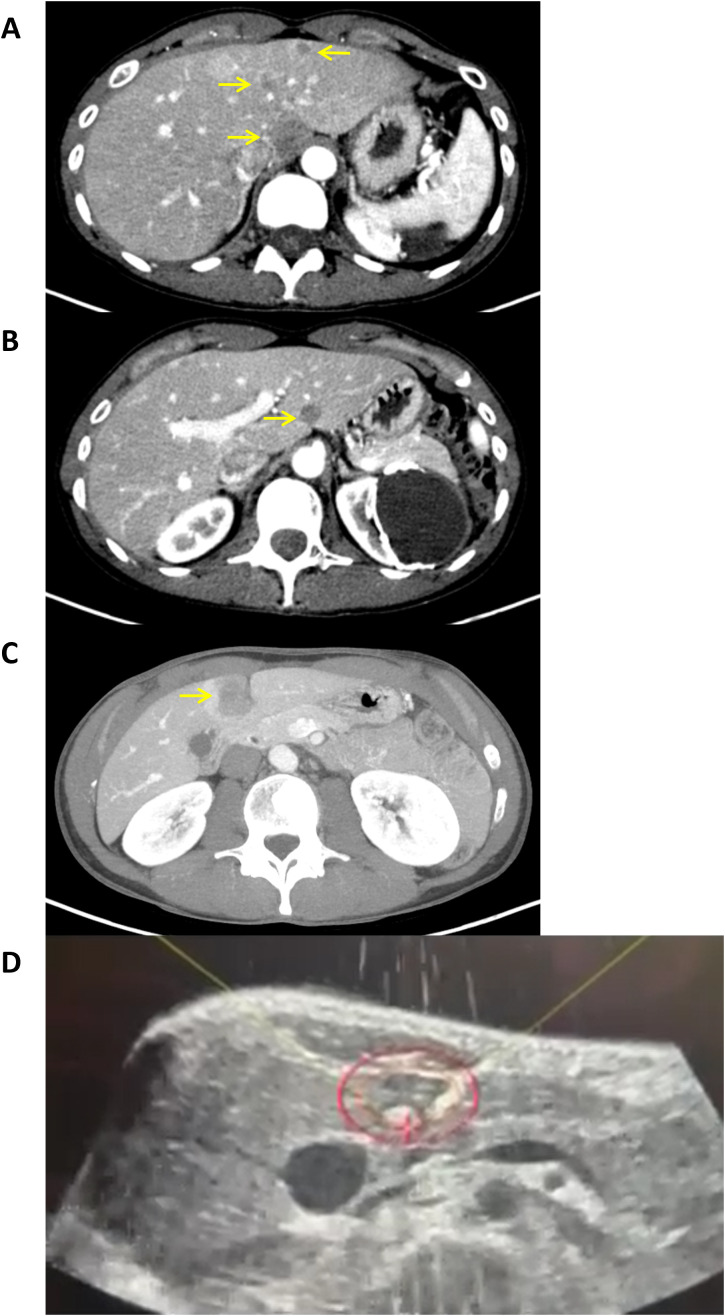
**(A–C)** Axial contrast-enhanced CT scans of liver before histotripsy treatment displaying multifocal breast cancer metastases in the left lobe. Target tumor lesions in segments 2, 3, and 4 are identified by arrows. **(D)** Intraprocedural ultrasound image of defined treatment volume within the red circle encompassing the hyperechoic targeted tumor. The focal point of the transducer is the red crosshair where the treatment effect is visible as an echogenic bubble cloud.

**Figure 2:**
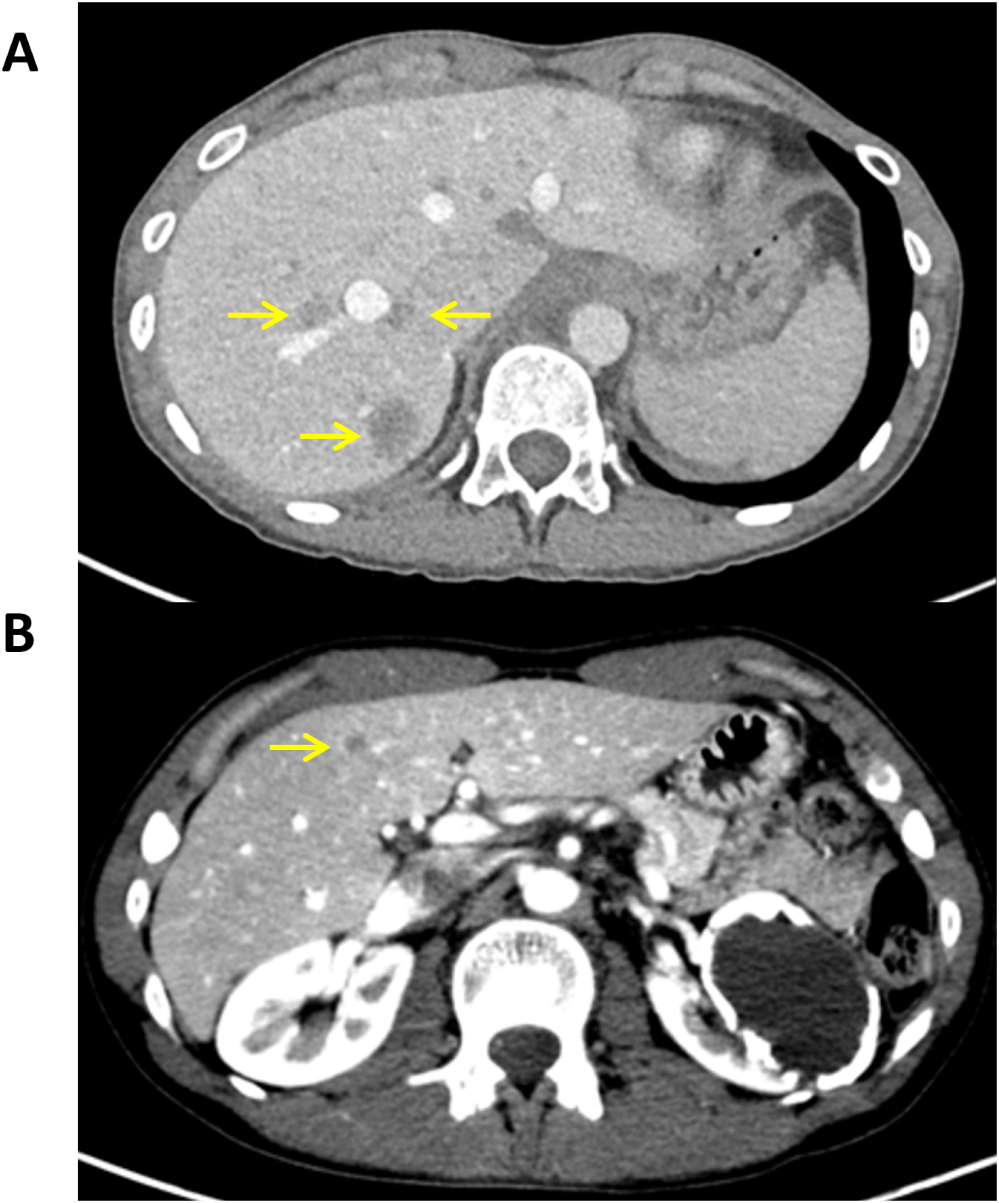
**(A, B)** Axial contrast-enhanced CT scans of liver before the second histotripsy treatment session displaying metastases throughout the right lobe of the liver (arrows).

**Figure 3:**
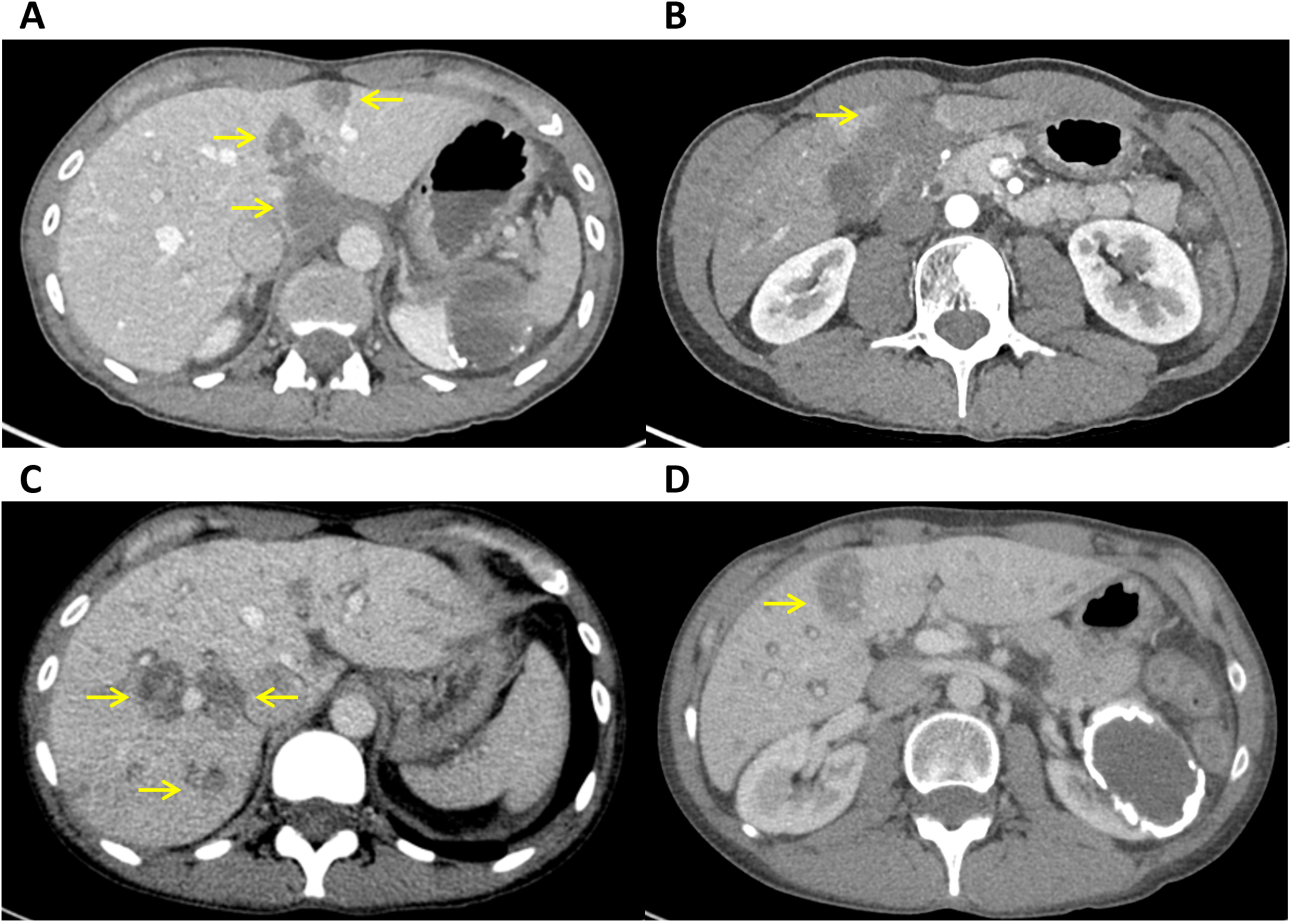
**(A, B)** Axial contrast-enhanced CT scans acquired within 24 hours after the first and **(C, D)** after the second histotripsy treatment session showing expected response in the treated lesions (yellow arrows).

After multidisciplinary review of the patient’s history, liver−directed histotripsy was recommended to treat her hepatic disease while maintaining her concurrent targeted therapy. Due to the extensive tumor burden present in the liver, a decision was made to stage the histotripsy treatments, by targeting the lesions in the left lobe first followed by the right lobe, provided the patient tolerated the initial histotripsy procedure well (**Figures 1**, **2**). She underwent the first histotripsy procedure in April 2025 targeting five lesions in the left lobe. On the day of the procedure, the patient presented with an ECOG performance status score of 0, normal liver function (alkaline phosphatase 54 U/L, ALT 28 U/L, AST 35 U/L, and bilirubin 0.3 mg/dL) and slightly elevated CA 15-3 36.6 U/mL. Histotripsy was performed with the Edison System (HistoSonics, Plymouth, Minnesota) as previously described (12). The procedure was performed under general anesthesia with left-sided double lumen intubation. The first five tumors were targeted using a handheld ultrasound device to optimize positioning and angulation of the treatment transducer. The planning treatment volume (PTV) included maximum diameters of 3.5, 2.35, 4.0, 2.5 and 1.85 cm for lesions located in segments 4a (deep), 4a (superficial), 4b, 3, and 2, respectively. To maximize treatment efficacy, a “surgical” margin was included to ensure adequate tumor coverage, considering breathing motion and microscopic extension (0.5 cm circumferential on average). Care was taken to prevent the PTV from directly overlapping with nearby organs at risk, such as the lung, bowel, and stomach. Five treatments were performed over 40 minutes, with the entire histotripsy procedure lasting around two and half hours. An example of the intraprocedural tumor targeting is presented in **Figure 1D**. Post-histotripsy CT imaging performed two hours later confirmed non−enhancing treatment zones corresponding to the planned targets (**Figures 3A, B**). Given the absence of peri-procedural complications or toxicities, the patient was discharged the same day, though she experienced post-procedure substernal pain that resolved within a few days. Two days after histotripsy, she received another dose of trastuzumab deruxtecan. At the end of April 2025, the patient experienced mild drug-induced pneumonitis that was treated with a steroid taper but continued the next scheduled dosing of trastuzumab deruxtecan.

**Figure 4:**
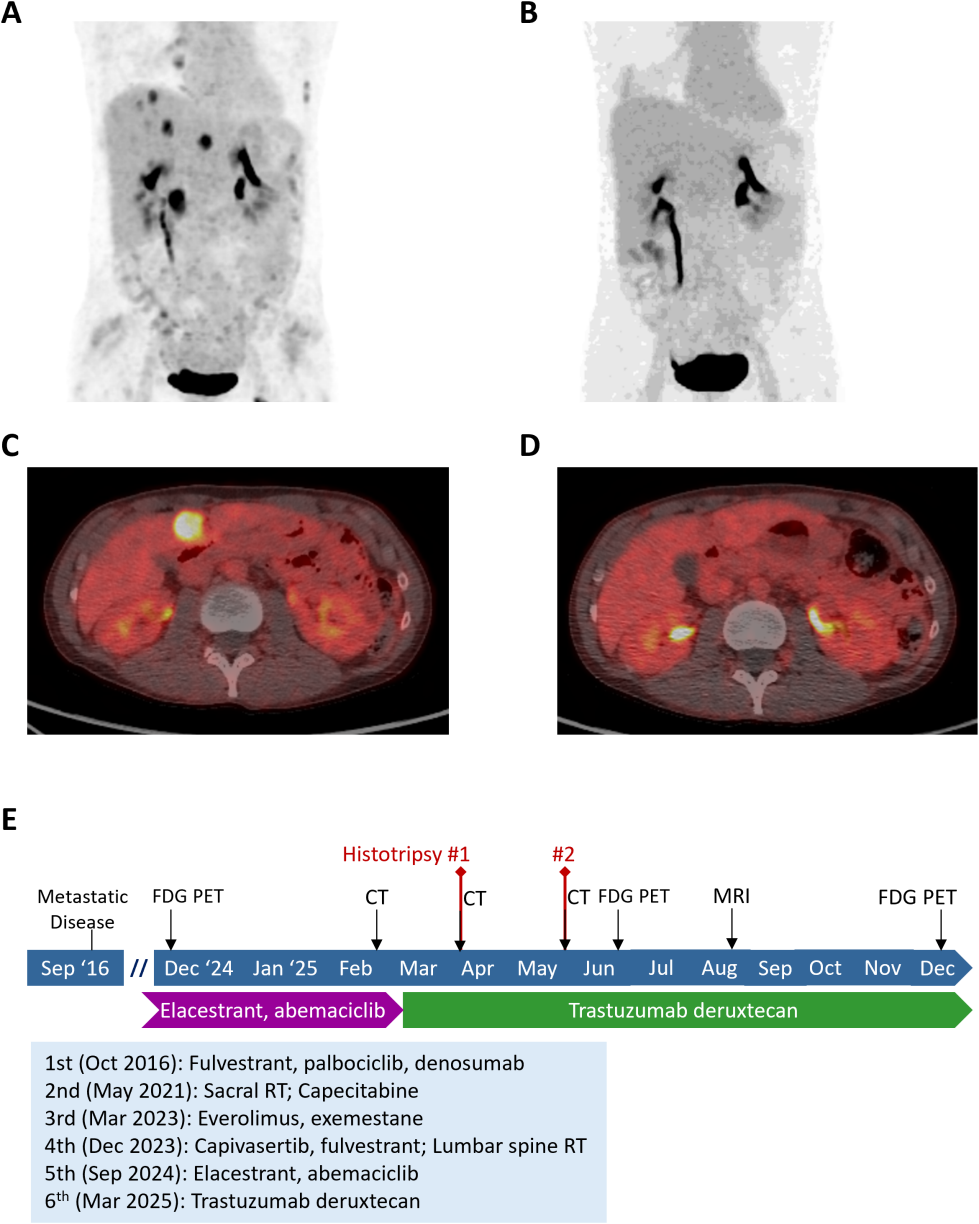
**(A, C)** Representative FDG PET-CT images of the liver prior to histotripsy and **(B, D)** 1 month after the second histotripsy treatment session. **(E)** A schematic time course of the entire case.

A second histotripsy procedure was performed in May 2025, this time targeting one lesion located in segment 4 and four additional tumors in the right lobe. On the day of the procedure, the liver function tests were within normal ranges (alkaline phosphatase 47 U/L, ALT 36 U/L, AST 36 U/L, bilirubin 0.4 mg/dL) and CA 15–3 had decreased to 25.3 U/mL (previously 36.6). The PTV maximum diameters were 2.5, 2.0, 2.5, 2.0, and 2.6 cm. Five treatments were performed over 27 minutes, with the entire procedure lasting approximately two hours. The patient experienced urine discoloration and mild post-procedural pain in the right upper quadrant of the abdomen but otherwise tolerated the procedure well. A CT scan was performed within a few hours (**Figures 3C, D**), and the patient was discharged the same day. The following day, alkaline phosphatase remained normal (56 U/L and 0.29 mg/dL, respectively) whereas both ALT and AST spiked post procedure (516 U/L and 483 U/L, respectively); however, the next dose of trastuzumab deruxtecan was given two days after histotripsy without any issue. Follow-up PET/CT imaging at the end of June 2025 showed absence of FDG−avid hepatic lesions, with significant overall metabolic regression when compared to prior FDG-PET imaging (**Figures 4A–D**). Liver function also normalized (alkaline phosphatase 98 U/L, ALT 31 U/L, AST 42 U/L, and bilirubin 0.23 mg/dL) and CA 15–3 decreased to within normal range (24.2 U/mL). In August 2025, MR imaging demonstrated expected post−treatment involution of the treated zones without internal nodular or rim enhancement to suggest the presence of viable tumors. The absence of FDG-avid hepatic lesions was again observed on subsequent PET/CT imaging in December 2025 along with CA 15-3 19.8 U/mL remaining within normal range. A timeline of the patient’s treatment course is shown in **Figure 4E**.

## Patient perspective

The patient described a high level of confidence in pursuing histotripsy after hearing the treating physician describe the technology, noting that it helped her feel reassured about the procedure’s potential to effectively treat her liver tumors. Both histotripsy sessions exceeded her expectations, resulting in successful destruction of ten liver metastases. She described the recovery as notably rapid, with only 24 hours of flu-like symptoms followed by mild abdominal discomfort that resolved within a week, allowing her to resume running seven days after treatment.

In describing her treatment decision, the patient emphasized that she sought a non-invasive, cutting-edge therapy to complement chemotherapy. She specifically contrasted her experience with expectations for traditional surgery, noting that her histotripsy recovery was “very manageable” when compared with that of surgical approaches which typically involve “weeks if not months of downtime.” This difference contributed to her belief that histotripsy aligned better with her personal goals, lifestyle, and desire to minimize disruption during treatment. The patient expressed strong satisfaction and gratitude for the care she received, stating she “could not be happier” with her decision and clinical team.

## Discussion

Although the management of patients with metastatic breast cancer remains extremely challenging, new image-guided therapeutic options such as histotripsy are emerging. Our case illustrates the potency of histotripsy in eradicating metastatic liver tumors in keeping with its FDA label indication. This patient with liver-dominant metastatic breast cancer had exhausted multiple lines of systemic targeted drug and liver directed therapy before being treated with histotripsy in a staged fashion to maximize treatment efficacy and minimize toxicity in a similar manner to radioembolization. Despite the extensive tumor burden as demonstrated on the pre-treatment FDG-PET/CT imaging, the treatment sessions were highly effective and well tolerated, consistent with results of previous clinical studies and real-world data of histotripsy.

Other loco-regional therapies including ablative and intraarterial therapies have been used to treat patients with metastatic breast cancer, but the main advantages of histotripsy are its completely noninvasive nature, lack of thermal deposition or radiation-induced effect, and the fact it does not prohibit concomitant use of systemic drug therapy. This latter aspect was fully exploited during the management of our patient as treatment with trastuzumab deruxtecan was not interrupted during the two sessions of histotripsy, allowing the patient to benefit from HER2 blockade while also being treated locally in the liver.

Another benefit of histotripsy is its ability to treat lesions near vascular or biliary structures without causing injury, as demonstrated in previous data and confirmed in our patient with widespread liver metastases. Data from the #HOPE4LIVER clinical trial and subsequent real world clinical practices report encouraging local tumor control and low major complication rates, supporting the use of histotripsy in appropriately selected liver tumors (13). The tumor burden of the patient with breast metastases in our case far exceeded what would have been considered appropriate for other ablative therapies given the number, location, and size of lesions. As a result, a decision was made to stage the procedure in a fashion that is usually reserved for intraarterial liver therapies such as chemoembolization or radioembolization where a segmental or lobar approach is often preferred to ensure complete disease coverage (14). Using histotripsy in this fashion opens the door to consider treating patients with larger tumor burdens than the more limited disease burden permitted in initial clinical trials. Staging the histotripsy procedures allowed the clinical team to preserve liver function within normal range, ensuring the continuation of concomitant systemic drug therapy. Ultimately, the patient responded in a dramatic fashion (**Figure 3**) illustrated by the complete disappearance of all FDG uptake throughout the liver lesions suggesting complete tumor cell death. Importantly, the patient also reported that she was able to maintain her normal activities of daily living while receiving combined therapy and experienced resolution of her cancer-related pain in the abdomen.

The present case report has several limitations. By definition, as a single-patient observation, the findings are not generalizable to the broader population of patients with metastatic breast cancer, particularly those with extensive hepatic tumor burden. Because the patient was receiving systemic therapy concurrently with histotripsy, the relative contribution of each treatment modality to the observed complete metabolic response cannot be determined with absolute certainty. The histotripsy procedures also require substantial logistical resources - including general anesthesia, extended procedure time, and careful treatment planning using imaging- which may initially limit accessibility and scalability in routine clinical practice. Additionally, finding a suitable acoustic window may be challenging at times thereby limiting the use of histotripsy for certain tumor locations. Finally, although our patient’s imaging findings are encouraging, the follow-up period remains relatively short making determination of long term benefit difficult to establish. This is expected since histotripsy has only been approved by the FDA for slightly more than 2 years. Nonetheless, our results reinforce the need for dedicated clinical studies with meaningful oncologic endpoints.

## Conclusion

Histotripsy is an exciting new therapy with significant advantages including its non-invasiveness, lack of thermal and ionizing effect and perhaps more importantly its ability to be combined with systemic therapies. A patient with liver-dominant metastatic breast cancer who experienced significant disease progression despite several lines of systemic therapy was treated with two staged sessions of histotripsy given the extensive tumor burden in the liver without interrupting systemic drug HER2 blockade which resulted in complete response by PET imaging at two and six months. Such results warrant further studies and indicate a potential new role for histotripsy in managing patients with extensive tumor burdens.

## Data Availability

The original contributions presented in the study are included in the article/supplementary material. Further inquiries can be directed to the corresponding author.
